# Assessment of Natural Radioactivity Levels and Radiation Hazards in Agricultural and Virgin Soil in the State of Kedah, North of Malaysia

**DOI:** 10.1155/2016/6178103

**Published:** 2016-11-14

**Authors:** Ghazwa Alzubaidi, Fauziah B. S. Hamid, I. Abdul Rahman

**Affiliations:** ^1^Physics Department, Faculty of Science, University of Malaya, Kuala Lumpur, Malaysia; ^2^Institute of Biological Science, Faculty of Science, University of Malaya, Kuala Lumpur, Malaysia; ^3^Centre for Research in Waste Management, Faculty of Science, University of Malaya, 50603 Kuala Lumpur, Malaysia; ^4^School of Applied Physics, Faculty of Science & Technology, Universiti Kebangsaan, Selangor, Malaysia

## Abstract

The activity concentrations of naturally occurring radionuclides ^226^Ra, ^232^Th, and ^40^K were determined in 30 agricultural and virgin soil samples randomly collected from Kedah, north of Malaysia, at a fertile soil depth of 0–30 cm. Gamma-ray spectrometry was applied using high-purity germanium (HPGe) gamma-ray detector and a PC-based MCA. The mean radioactivity concentrations of ^226^Ra, ^232^Th, and ^40^K were found to be 102.08 ± 3.96, 133.96 ± 2.92, and 325.87 ± 9.83 Bq kg^−1^, respectively, in agricultural soils and 65.24 ± 2.00, 83.39 ± 2.27, and 136.98 ± 9.76 Bq kg^−1^, respectively, in virgin soils. The radioactivity concentrations in agricultural soils are higher than those in virgin soils and compared with those reported in other countries. The mean values of radium equivalent activity (Ra_eq_), absorbed dose rates *D* (nGy h^−1^), annual effective dose equivalent, and external hazard index (*H*_ex_) are 458.785 Bq kg^−1^, 141.62 nGy h^−1^, and 0.169 mSv y^−1^, respectively, in agricultural soils and 214.293 Bq kg^−1^, 87.47 nGy h^−1^, and 0.106 mSv y^−1^, respectively, in virgin soils, with average *H*_ex_ of 0.525. Results were discussed and compared with those reported in similar studies and with internationally recommended values.

## 1. Introduction

Humans are exposed to natural radioactivity at different levels depending on natural radioactive elements present in each area; as such, researchers investigated the natural environmental radiation and radioactivity in soils to conduct background checks and detect environmental radioactivity [1]. The levels of radioactivity can be used to assess public dose rates and radioactive contamination and predict changes in environmental radioactivity caused by nuclear accidents, industrial activities, and other human activities [2].

Potassium-40, uranium-238, and thorium-232 and their decay products are important natural elements that contribute to a large part of the radiation dose received by humans; thus far, approximately 60 abundantly distributed radionuclides have been identified. Radionuclides are encountered in terrestrial strata (soil or rocks) or lakes and water bodies (ocean, sea, or lakes) and can be easily accumulated into the food chain [3].

Specific levels of terrestrial environmental radiation are related to the geological composition of each lithologically separated area and to the contents of thorium (Th), uranium (U), and potassium (K) in rocks from which soils originate. Soils are categorized into several types depending on their physical and chemical properties. Many studies conducted worldwide showed that ^238^U, including its decay products in soils and rocks, and ^232^Th in monazite sands are the main sources of high natural background radiation [4].

Natural environmental radiation and radioactivity in soils have gained considerable research interest because humans are exposed to natural radioactivity at different levels depending on natural radioactive minerals present in each region worldwide [1].

Radionuclides in phosphate rocks can enter the environment through different mechanisms, such as use of phosphogypsum for building materials and for agriculture or fertilization of agricultural lands. Direct fallout from the atmosphere on the vegetation is the primary source of radiation contamination. Fission product ^137^C is strongly absorbed and maintained by soil particles, similar to natural radionuclides, which are widely distributed at different soil depths. Therefore, knowledge on radionuclide distribution in soils is essential to control health risks to the affected population.

In this study, the concentration of radioactivity and the radiation dose from agricultural soils of rice farms and virgin soils in Kedah, Malaysia, were analyzed. Results can be used to determine public dose rates, assess the performance of epidemiological studies, and maintain reference data to ascertain changes in environmental radioactivity caused by nuclear, industrial, and other human activities.

## 2. Experimental Procedures

### 2.1. Location of Study Area

**Figure 1:**
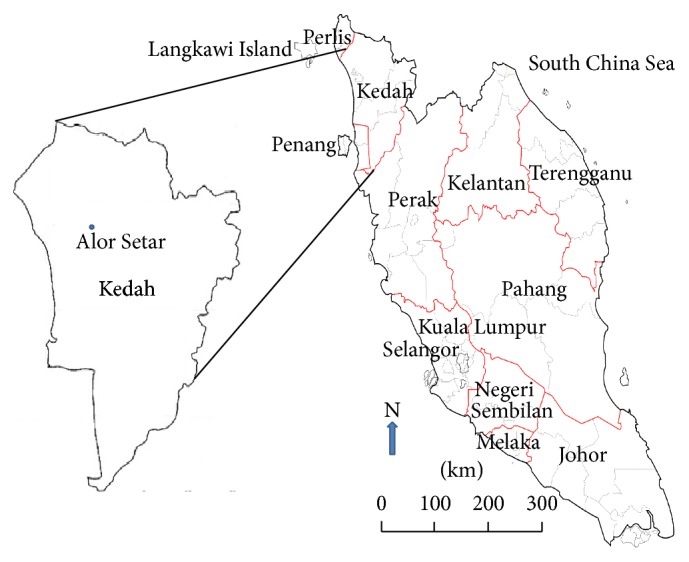
Map of the area studied.

Surface soil samples were randomly collected from different locations of fertile soil in Kedah in the northwestern part of Peninsular Malaysia 6°7′6.2400′′N and 100°22′6.4560′′E (Figure 1) and used to measure natural radioactivity in soil. Kedah covers an area of 9,425 sq.  km, and its agriculture industry is largely dedicated to industrial crops. About 518 ha of land in the state is utilized for agriculture, 108 ha is covered by rubber trees, 84 ha is planted with oil palms, and 112 ha is used for cultivating rice. The mainland has a relatively flat terrain and is widely used to grow rice. The average annual rainfall in Kedah ranges from 2032 mm to 2540 mm.

Vast agricultural areas in the state of Kedah and intensive use of phosphate fertilizers for reclaiming farmland have impelled researchers to investigate the concentration levels of radionuclides in these areas and compare them with those of virgin soil. The levels of radioactivity concentration of radionuclides in agriculture and virgin soil samples provide useful information for monitoring of environmental radiation contamination.

### 2.2. Samples Collection and Preparation

Thirty samples were collected at a depth of 0–30 cm from rice farms and virgin soil to measure natural radioactivity. The samples were dried at 100°C for 24 h in an oven and constant dry weights were obtained to determine moisture content [23].

The dried samples were crushed into fine powder by using a soil blender. The powdered samples were sieved through 100-micron mesh to keep uniform grain size and obtain affine-grained homogenous soil samples for measurements [24]. About 500 g of the homogenous soil sample was packed and sealed in an air-tight Marinelli beaker and stored for 4 weeks before gamma-ray analysis; this incubation period allows  ^226^Ra and its short-lived progenies to achieve secular equilibrium [25, 26].

Gamma spectrometry analysis was performed using a gamma-ray spectrometer with a p-type coaxial HPGe *γ*-ray spectrometer and a p-type coaxial ORTEC, GEM-25 HPGe gamma-ray detector with 57.5 mm diameter and 51.5 mm thick crystals.

The detector was set under the following conditions: operating voltage, +2800 V; relative efficiency, 28.2%; energy resolution, 1.67 keV; and full width at half maximum, 1.33 MeV. The detector was coupled with ^60^C emission and 16 k Multichannel Analyzers for data acquisition. Genie 2000 software from Canberra was used to analyze the spectra. The detector was covered by a cylindrical lead shield with a fixed bottom and a movable cover to reduce the interference of background radiation from terrestrial and extraterrestrial sources in the measured spectrum.

An empty Marinelli beaker was counted in the same way to remove the background radiation from the samples. After the measurement, the background radiation was subtracted to determine naturally occurring background distribution in the environment around the detector [6].

Energy calibration and relative efficiency calibration of the spectrometer were performed using Marinelli calibration sources containing the following: ^210^Pb (46.54 keV), ^241^Am (59.541 keV), ^109^Cd (88.040 keV), ^57^C (122.061 and 136.474 keV), ^123m^Te (159.00 keV), ^203^Hg (279.195 keV), ^113^Sn (391.698 keV), ^85^Sr (514.007 keV), ^137^Cs (661.657 keV), ^88^Y (898.042 and 1836.063 keV), and ^60^Co (1173.22 and 1332.492 keV). The calibration source with an initial activity of 5.109 *μ*Ci was obtained from Isotope Products Laboratories (Valencia, CA 91355, USA).

Each sample and background data were counted for 86400 s. Gamma spectroscopy was used to determine the activities of ^238^U, ^232^Th, and ^40^K.

The specific activity of ^226^Ra was assessed from gamma-ray lines of  ^214^Pb at 351 keV and ^214^Bi at 609.3 and 1764.5 keV, while the specific activity of ^232^Th had been evaluated from gamma-ray lines of ^228^Ac at 338.4, 911.1, and 968.9 keV, ^212^Pb at 238.63 keV, and ^208^Tl at 583.19 keV. The specific activity of ^40^K was directly determined from its gamma-ray line at 1460.8 keV (Table 1).

## 3. Results and Discussion

### 3.1. The Activity Concentration

Radioactivity concentration was measured using a gamma-ray spectrometer. The radioactivity of natural radionuclides, namely, uranium and thorium series, as well as ^40^K, was investigated in soil samples collected from Kedah. The primordial radionuclides of  ^226^Ra, ^232^Th, and ^40^K were the three most important detected in the zone [27, 28].

The radioactivity concentrations of these radionuclides were calculated using the following formula [29]:

(1) $$\begin{array}{c} A=\frac{N}{P\gamma \times \varepsilon \times W}\left(\;Bq\;\;{kg}^{-1}\;\right), \end{array}$$

where *A* = (Bq kg^−1^), *N* is net counts per second (CPS) = (sample CPS – background CPS), *Pγ* is intensity of the radionuclide, *E* is efficiency in %, and *W* is weight of sample in gram.

The activity concentrations in 30 soil samples that were determined using HPGe detector are reported in (Table 2). The measured activity concentration of ^226^Ra in agricultural soil samples ranged from 58.93 ± 1.80 Bq kg^−1^ to 166.55 ± 6.66 Bq kg^−1^, with a mean value of 102.08 ± 3.96 Bq kg^−1^. The concentration of ^232^Th ranged from 87.98 ± 1.35 Bq kg^−1^ to 180.45 ± 3.15 Bq kg^−1^, with an average value of 133.96 ± 2.92 Bq kg^−1^. The activity of ^40^K in agricultural soil samples ranged from 202.2 ± 11.72 Bq kg^−1^ to 529.17 ± 10.19 Bq kg^−1^, with an average value of 325.87 ± 9.83 Bq kg^−1^.

The activity concentration of ^226^Ra in virgin soil samples varied from 45.11 ± 2.44 Bq kg^−1^ to 111.4 ± 1.3 Bq kg^−1^, with a mean value of 65.24 ± 2.00 Bq kg^−1^. The activity concentration of ^232^Th ranged from 51.83 ± 1.18 Bq kg^−1^ to 127.35 ± 6.03 Bq kg^−1^, with an average value of 83.39 ± 2.27 Bq kg^−1^. The concentration of  ^40^K radionuclides in soil samples ranged from 99.2 ± 12.1 Bq kg^−1^ to 172.85 ± 7.71 Bq kg^−1^, with an average value of 136.98 ± 9.76 Bq kg^−1^.

The recommended reference levels of ^226^Ra, ^232^Th, and ^40^K are 35, 30, and 400 Bq kg^−1^, respectively, as listed in the world average concentrations published by UNSCEAR (2000). The average concentrations of ^226^Ra and ^232^Th obtained in the present study are higher than the recommended reference levels. The mean concentrations of the natural radioactivity of virgin and agricultural soils were also compared with the range and average of the natural radioactivity concentration levels reported in other studies (Table 3). The mean concentrations of ^226^Ra, ^232^Th, and ^40^K in virgin and agricultural soils in the present study are higher than those reported by Ahmad et al. [7] and Saleh et al. [6] studies which was carried out by the first researcher to assess the concentration radioactivity levels in agricultural areas of palm oil and bananas of Kedah.

The results were also compared with those reported in studies conducted in other countries (Table 6). The mean activity concentrations of natural radioactivity of ^226^Ra, ^232^Th, and ^40^K in agricultural soil samples in the present study are higher than those reported in agricultural soils of India, Pakistan, Algeria, Egypt, Thailand, and Greece. Phosphate fertilizers are extensively applied in the farmlands of rice; therefore, the activity concentration of  ^226^Ra was enhanced in these farmlands. The enhancement in the radioactivity concentration of  ^226^Ra could be attributed to fertilization with phosphate rocks, which contain substantial amounts of ^238^U, ^226^Ra, ^232^Th, and ^226^Ra decay products; this phenomenon results in the high activity of ^40^K in soil [30].

High radioactivity concentrations in the soil of the present studied area were also reported in previous studies by Ahmad et al. [7, 31] but were lower than those of ^226^Ra, ^232^Th, and ^40^K reported by Almayahi et al. [5] and Ahmad et al. [7, 31], as well as by Saleh et al. [6] in virgin soil samples.

The mean radioactivity concentrations of ^232^Th and  ^226^Ra in virgin soil in the present study are higher than those reported by UNSCEAR [2] (Table 7), whereas the mean value of ^40^K is slightly lower than that reported worldwide, except for Japan and Egypt.

Variations in the radioactivity concentrations in soils of various locations worldwide depend on the geographical and geological conditions of the zone and the extent of fertilizer utilized in farmland [32, 33].

### 3.2. Radiological Hazard Assessment

#### 3.2.1. Assessment of Radium Equivalent (Ra_eq_)

Gamma-ray radiation hazards caused by specific radionuclides of ^226^Ra,^232^Th, and ^40^K were evaluated using different indices. Ra_eq_, which is the radium equivalent activity, is the most widely used radiation hazard index [34, 35]. Ra_eq_ is the weighted sum of activities of the three radionuclides based on the supposition that 370 Bq kg^−1^ ^226^Ra, 259 Bq kg^−1^ ^232^Th, and 481 Bq kg^−1^ ^40^K produce the same gamma-ray dose rate [36]. Ra_eq_ is given by [37]

(2) $$\begin{array}{c} Ra_{eq}\left(\;Bq\;{kg}^{-1}\;\right)=CRa+1.43CTh+0.077CK, \end{array}$$

where *C*Ra, *C*Th, and *C*K are the activity concentrations of ^226^Ra, ^232^Th, and ^40^K (in Bq kg^−1^), respectively.

To keep the annual radiation dose below 1.5 m Gy y^−1^, the maximum value must be less than 370 Bq kg^−1^ [8].

As shown in Table 5, Ra_eq_ of agricultural soil samples was within the range of 274.75–819.86 Bq kg^−1^, with a mean value of 458.785 Bq kg^−1^, which exceeds the permissible limit (370 Bq kg^−1^) recommended by the Organization for Economic Cooperation and Development [38]. The mean of Ra_eq_ in the virgin soil was found to be 214.293 Bq kg^−1^, which is within the permissible limit.

The permissible limit of Ra_eq_ in building materials must be <370 Bq kg^−1^, which is equal to an annual dose of 1.5 mSv y^−1^ [39, 40].

#### 3.2.2. Absorbed Dose Rate in Air (*D*)

According to the guidelines provided by UNSCEAR [2], the absorbed gamma dose rate *D*_*R*_ (nGy h^−1^) in air was determined at 1 m above the ground surface to ensure uniform distribution of radionuclides. This parameter can be used to assess any radiological hazard and radiation exposure from radionuclides in the soil; the absorbed dose rate was calculated using the following formula [41]:

(3) $$\begin{array}{c} D_{R}\left(\;nG\;h^{-1}\;\right)=0.427CRa+0.623CTh+0.043CK, \end{array}$$

where *D*_*R*_ is the dose rate in nGy h^−1^ and *C*Ra, *C*Th, and *C*K are the activity concentrations (Bq kg^−1^) of radium (^226^Ra), thorium (^232^Th), and potassium (^40^K), respectively.

The absorbed dose rate indicates the received dose outdoors from radiation emitted by radionuclides in environmental materials. Determination of this rate is the main step for evaluating health risk, and this parameter is expressed in gray.

Table 5 shows the absorbed dose rate calculated from the radioactivity concentrations of ^226^Ra, ^232^Th, and  ^40^K in agricultural and virgin soil samples.

The absorbed dose rate in agricultural soil ranged from 91.34 nGy h^−1^ to 207.34 nGy h^−1^, with a mean value of 141.62 nGy h^−1^, which is higher than the global mean value of 60 nGy h^−1^ established by UNSCEAR [2].

The average value of the absorbed dose rate *D* (nGy h^−1^) of agricultural soils in the present study is higher than those reported in other countries (Table 6). The absorbed gamma dose rate in virgin soil samples ranged from 60.71 nGy h^−1^ to 129.04 nGy h^−1^, with an average value of 87.47 nGy h^−1^, which is higher than the mean values reported in United States, Japan, Egypt, Poland, and Switzerland (Table 7) and the value recommended by UNSCEAR [2].

#### 3.2.3. The Annual Effective Dose Rate

Annual effective dose should be calculated to assess the health effects of the absorbed dose by using a conversion coefficient (0.7 Sv Gy^−1^) to transform absorbed dose in air to the effective dose received by humans, with an outdoor occupancy factor (0.2), which is equivalent to an outdoor occupancy of 20% and 80% for the indoors [38, 42]. This factor is suitable for determining the pattern of life in the studied area. Annual effective dose rate (AEDR, in mSv y^−1^) received by the population can be calculated using [43, 44]

(4) Annual  effective  dose  rate  mSv  y−1=Absorbed  dose  nGy h−1×8760 h·yr−1×0.7×103 mSv/10−9×0.2nGy−1=D×1.2264×10−3mSv y−1,

where *D* (nG/h) is the total air absorbed dose rate in the outdoors; 8760 h is the number of hours in one year; 0.2 is the outdoor occupancy factor; 0.7 Sv Gy^−1^ is the conversion coefficient from absorbed dose in air to effective dose received by adults; 10^−6^ is the conversion factor between nano- and millimeasurements.

The estimated annual effective dose in the agricultural soil samples ranged from 0.112 mSv y^−1^ to 0.254 mSv y^−1^, with an average value of 0.169 mSv y^−1^, whereas that for virgin soil samples ranged from 0.073 mSv y^−1^ to 0.158 mSv y^−1^, with an average value of 0.106 mSv y^−1^. As shown in (Table 5), the worldwide average annual effective dose is approximately 0.5 mSv y^−1^ [2]. Thus, the present average annual effective dose rates are within the average values reported worldwide.

Indoor dose rates were not evaluated because data on average buildup of radon gas in the indoor atmosphere were not available.

#### 3.2.4. External Hazard Index (*H*_ex_)

The external hazard index for samples under investigation was calculated using the equation defined by [33].

(5) $$\begin{array}{c} H_{ex}=\frac{CRa}{370}+\frac{CTh}{259}+\frac{CK}{4810}\leq 1, \end{array}$$

where *C*Ra, *C*Th, and *C*K are the activity concentrations of ^226^Ra, ^232^Th, and ^40^K in (Bq kg^−1^), respectively. The maximum value of *H*_ex_ equal to unity corresponds to the upper limit of Ra_eq_ (370 Bq kg^−1^).

The calculated values of *H*_ex_ for agricultural soil samples ranged from 0.552 to 1.252, with a mean value 0.859, whereas those for virgin soil samples ranged from 0.362 to 0.789, with an average value 0.525 (Table 5). The value of *H*_ex_ must be lower than unity to keep the radiation hazard insignificant. These values are less than the limit (*H*_ex_ less than or equal to one) established by the European Commission on Radiation Protection (1999) [13]; hence, terrestrial soils from the study area present low radiation exposure for people and can be used as a construction material without posing any significant radiological threat to the general population.

#### 3.2.5. Gamma Index (*I*_*γ*_)

Gamma index (*I*_*γ*_) proposed by the European Commission has been calculated from the activity concentrations of ^226^Ra, ^232^Th, and ^40^K in soil samples using the following formula [13]:

(6) $$\begin{array}{c} I_{\gamma }=\frac{A_{Ra}}{300}+\frac{A_{Th}}{200}+\frac{A_{K}}{3000}\leq 1, \end{array}$$

where *A*_Ra_, *A*_Th_, and *A*_K_ are the activity concentrations (Bq kg^−1^) of radium (^226^Ra), thorium (^232^Th), and potassium (^40^K), respectively.

Values of index *I*_*γ*_ ≤ 2 correspond to an absorbed gamma dose rate of 0.3 mSv/year, whereas 2 < *γ* ≤ 6 corresponds to an absorbed gamma dose rate of 1 mSv/year [13, 45], andmaterials with *I*_*γ*_ > 6 correspond to dose rates higher than 1 mSv/year, which is the highest dose rate value recommended for the population [13].

Therefore, the annual effective dose that can be delivered by the soil as building materials in this study is lower than the annual effective dose constraint of 1 mSv/year.

The calculated values of agricultural and virgin soil samples are presented in Tables 2 and 5; gamma indices of agricultural soil are varying from 0.7 to 1.6, with a mean value of one that is found to be higher than the limit of 0.5, while those found in the virgin soils are varying from 0.4 to 1 with a mean value of 0.59. It is observed that the mean values of agricultural and virgin soils did not exceed the recommended upper limit (Table 4). Therefore, the annual effective dose that can be delivered by the soil as building materials in this study is lower than the annual effective dose constraint of 1 mSv/year.

Moreover, the gamma-index values of our study are comparable with results of various studies around the world (Table 6).

## 4. Conclusion

Gamma spectrometry was used to measure the radioactivity concentration of 30 agricultural and virgin soil samples collected from Kedah Region, north of Malaysia. Results showed that the mean activity concentrations of ^226^Ra, ^232^Th, and ^40^K are 102.08 ± 3.96, 133.96 ± 2.92, and 325.87 ± 9.83 Bq kg^−1^, respectively, in agricultural soil samples and 65.24 ± 2.00, 83.39 ± 2.27, and 136.98 ± 9.76 Bq kg^−1^ in virgin soil samples. The measured values are higher than those reported in other soils worldwide. The average activity concentrations of ^226^Ra and ^232^Th (Bq kg^−1^) in virgin and agricultural soils are higher than the world recommended values UNSCEAR [2]. However, the average activity concentration of ^40^K is below the recommended values in both soil types. No ^137^Cs activity concentration was found in any of the samples from this district, indicating the absence of artificial radionuclide fallout from any nuclear accidents.

The mean value of gamma absorbed dose in air outdoors are within the range of 91.34–207.34 nGy h^−1^, with a mean value of 141.62 nGy h^−1^, for agricultural soils and within 60.71–129.04 nGy h^−1^, with an average of 87.47 nGy h^−1^, in virgin soil; these values are higher than the global average value of 60 nGy h^−1^ UNSCEAR [2] in both soil types.

The average annual effective dosages from agricultural and virgin soil samples are also lower than the global average values.

The value of Ra_eq_ activity concentrations for agricultural and virgin soil samples is less than 370 Bq kg^−1^, with the mean value exceeding the permissible limit recommended by the Organization for Economic Cooperation and Development (NEA-OECD report) [38] in agriculture soil samples.

The mean value of the external hazard index *H*_ex_ of the study area is found to be within the recommended safe levels (*H*_ex_ less than or equal to one). The obtained results of gamma index (*I*_*γ*_) are within the recommended safety limits of European Commission (1999).

This study established a map of baseline information for future studies on radiation levels and radionuclide distribution in the environment of Kedah. The results of the study serve as a reference for future assessment.

## Figures and Tables

**Table 1:** Gamma-ray energy and emission rate for ^238^ U, ^232^Th, and ^40^K radionuclides.

Element	Nuclide	Half-life	Gamma-ray energy *E*_*g*_ (keV)	Emission rate	Sources/origin
^238^U	^214^Pb	26.8 min	351	35.8	^238^U (^226^Ra) series
	^214^Bi	19.9 min	609.3	45.4	^238^U (^226^Ra) series
			1764.5	15.3	

^232^Th	^228^Ac	6.15 h	338.4	11.4	^232^Th series
			911.1	25.8	
			968.9	17.4	
	^212^Pb		238.63	46.6	
	^208^Tl		583.19	85.0	

^40^K	^40^K	1.28 × 10^9^ yr	1460.8	10.7	Primordial

**Table 2:** The activity concentrations of  ^226^Ra, ^232^Th, and ^40^K (Bq kg^−1^) in agricultural and virgin soil samples collected from Kedah soil of north Malaysia.

Sample number	Activity concentrations (Bq kg^−1^)			*D* (nGy h^−1^)	AEDE (mSv y^−1^)	Ra_eq_ (Bq kg^−1^)	*H*_ex_	*I*_*γ*_
	^226^Ra	^232^Th	^40^K					
Agricultural soil samples								
S-A1	67.05 ± 4.07	121.56 ± 8.35	357.64 ± 12.03	118.76	0.145	394.7	0.724	0.9
S-A2	166.55 ± 6.66	180.45 ± 3.15	513.34 ± 1.61	207.34	0.254	819.86	1.252	1.62
S-A3	114.92 ± 4.60	150.61 ± 2.44	401.26 ± 16.05	160.79	0.197	669.26	0.974	1.26
S-A4	77.47 ± 1.90	95.83 ± 1.18	203.09 ± 6.63	102.14	0.125	275.05	0.621	0.8
S-A5	90.94 ± 3.36	112.29 ± 1.8	330.93 ± 11.53	123.63	0.151	506.33	0.746	0.97
S-A6	77.97 ± 3.12	95.55 ± 1.96	202.2 ± 11.72	102.16	0.125	274.75	0.621	0.8
S-A7	85.06 ± 1.92	134.92 ± 2.02	305.73 ± 1.06	133.53	0.163	378.48	0.814	1
S-A8	102.42 ± 4.78	155.71 ± 2.6	219.63 ± 9.90	150.52	0.184	338.5	0.923	1
S-A9	104.48 ± 4.18	167.6 ± 0.4	236.76 ± 9.37	159.36	0.195	358.85	0.978	1.26
S-A10	115.91 ± 6.24	135.91 ± 2.74	529.17 ± 10.19	157.702	0.193	717.72	0.947	1.24
S-A11	58.93 ± 1.80	87.98 ± 1.35	263.32 ± 5.63	91.34	0.112	299.5	0.552	0.7
S-A12	87.68 ± 3.38	98.08 ± 3.24	351.53 ± 21.65	114.4	0.140	370.53	0.688	0.8
S-A13	140.94 ± 2.96	174.13 ± 2.63	206.96 ± 21.29	178.9	0.219	375.165	1.096	1.3
S-A14	110.6 ± 9.45	143.31 ± 8.89	523.92 ± 1.03	159.5	0.195	718.95	0.959	1.24
S-A15	130.4 ± 1.0	155.5 ± 1.1	242.7 ± 7.9	164.28	0.201	384.14	1.003	1.28

Virgin soil samples								
S-V16	62.8 ± 1.4	75.2 ± 1.5	101.3 ± 13.5	78.65	0.096	173.13	0.481	0.6
S-V17	75.2 ± 1.4	80.4 ± 1.3	158.3 ± 15.3	89.90	0.110	231.66	0.545	0.7
S-V18	72.7 ± 1.6	104.7 ± 2.1	156.5 ± 9.0	103.34	0.126	283.22	0.631	0.6
S-V19	65.3 ± 1.4	67.3 ± 1.4	197.5 ± 13.4	79.05	0.096	246.3	0.477	0.6
S-V20	56.6 ± 1.2	77.7 ± 1.3	99.2 ± 12.1	78.06	0.095	166.4	0.473	0.6
S-V21	111.4 ± 1.3	120.2 ± 1.4	119.5 ± 14.0	129.04	0.158	255.09	0.789	1
S-V22	87.59 ± 3.90	94.2 ± 2.07	126.62 ± 8.64	120.63	0.147	225.6	0.626	0.6
S-V23	57.8 ± 1.3	63.2 ± 1.3	172.2 ± 9.6	72.05	0.088	217.57	0.434	0.5
S-V24	60.73 ± 2.43	70.71 ± 1.24	144.8 ± 5.45	76.80	0.094	273.34	0.467	0.6
S-V25	61.4 ± 1.4	65.3 ± 1.3	103.9 ± 7.5	72.13	0.088	169.48	0.438	0.5
S-V26	72.74 ± 5.13	127.35 ± 6.03	172.85 ± 7.71	117.72	0.144	260.6	0.722	0.7
S-V27	47.37 ± 1.9	51.83 ± 1.18	163.09 ± 6.63	59.98	0.073	195.23	0.362	0.4
S-V28	48.6 ± 1.92	105.73 ± 1.06	145.73 ± 1.06	92.38	0.113	206.27	0.569	0.5
S-V29	53.3 ± 1.2	88.2 ± 9.4	90.2 ± 9.4	81.65	0.100	160.68	0.502	0.6
S-V30	45.11 ± 2.44	58.91 ± 1.61	103.11 ± 13.16	60.71	0.074	149.83	0.370	0.4

**Table 3:** The mean activity concentrations of natural radioactivity of agriculture and virgin soils in the present study were compared with those from similar investigations performed in other countries.

Region/country	Concentration in soil (Bq kg^−1^)						Reference
	^226^Ra		^232^Th		^40^K		
	Mean	Range	Mean	Range	Mean	Range	
Virgin soil							
Malaysia (Penang)	396		165		835		Almayahi et al. [5]
Malaysia (Pontian)	37		53		293		Saleh et al. [6]
Malaysia (Kedah)	51.06		78.44		125.66		Ahmad et al. [7]
Malaysia	65.24	45.11–111.4	83.39	51.83–127.35	136.98	99.2–172.8	The present study

Agriculture soil							
Malaysia (Kedah)	80.63		116.87		200.66		Ahmad et al. [7]
India	64		93		124		Singh et al. [8]
Pakistan	30		56		602		Tufail et al. [9]
Algeria	53.2		50.03		311		Boukhenfouf and Boucenna [10]
Egypt	43	5.7–140	54	9–139	183	22–319	Issa [11]
Thailand	43	11–78	51	7–120	230	7–712	UNSCEAR [2]
Malaysia	66	49–86	82	63–110	310	170–430	UNSCEAR [2]
Greece	16 ± 6	12–26	55 ± 14	39–72	305 ± 59	222–376	Ioannides et al. [12]
Malaysia	102.08 ± 3.96	58.93–166.55	133.96	87.98–180.45	325.87	202.2–529.17	The present study

**Table 4:** Gamma-index (*I*_*γ*_) values proposed by the European Commission (1999) taking in to account typical way and amounts in which the material is used in a building [13].

Dose criterion	0.3 mSv y^−1^	1 mSv y^−1^
Materials used in bulk amounts	*I*_*γ*_ ≤ 0.5	*I*_*γ*_ ≤ 1
For example, bricks superficial and other materials with restricted use: tiles, boards, and so forth	*I*_*γ*_ ≤ 2	*I*_*γ*_ ≤ 6

**Table 5:** Range and mean value of activity concentrations of ^226^Ra, ^232^Th, and ^40^K (in Bq kg^−1^), Ra equivalent Ra_eq_ (Bq kg^−1^), absorbed dose rates *D* (nGy h^−1^), external hazard index (*H*_ex_), annual effective dose rates, AEDE, (mSv y^−1^) in soil samples of Kedah.

	Sample	Mean	Maximum	Minimum
Radionuclides				
^226^Ra	Agricultural soil	102.08 ± 3.96	166.55 ± 6.66	58.93 ± 1.80
	Virgin soil	65.24 ± 2.00	111.4 ± 1.3	45.11 ± 2.44
^232^Th	Agricultural soil	133.96 ± 2.92	180.45 ± 3.15	87.98 ± 1.35
	Virgin soil	83.39 ± 2.27	127.35 ± 6.03	51.83 ± 1.18
^40^K	Agricultural soil	325.87 ± 9.83	529.17 ± 10.19	202.2 ± 11.72
	Virgin soil	136.98 ± 9.76	172.85 ± 7.71	99.2 ± 12.1
Radiological hazard				
*D* (nGy h^−1^)	Agricultural soil	141.62	207.34	91.34
	Virgin soil	87.47	129.04	60.71
AEDE (mSv y^−1^)	Agricultural soil	0.169	0.254	0.112
	Virgin soil	0.106	0.158	0.073
Ra_eq_ (Bq kg^−1^)	Agricultural soil	458.785	819.86	274.75
	Virgin soil	214.293	283.22	149.83
*H*_ex_	Agricultural soil	0.859	1.252	0.552
	Virgin soil	0.525	0.789	0.362
Gamma index *I*_*γ*_	Agricultural soil	1.07	1.62	0.7
	Virgin soil	0.59	1	0.4

**Table 6:** Average hazard indices of the primordial radionuclides in the worldwide agricultural soils.

Location	*D* (nGy/h)	*D*_eff_ (mSv/y)	*H*_ex_	*I*_*γ*_	Reference
Vietnam	71.72	0.54	0.43	—	Huy et al. [14]
India	97.47	0.12		—	Mehra and Singh [15]
Saudi Arabia	23.3	0.14	0.13	—	Alaamer [16]
Malaysia	202.04	0.23	1.19	—	Musa et al. [17]
Jordan	51.50	0.06	0.28	—	Al-Hamarneh and Awadallah [18]
Pakistan	68.83	0.34	0.39	0.14	Rafique et al. [19]
India	90.1	0.11	0.53	0.71	Zubair et al. [20]
Egypt (Rashid)	118.36	145.16	0.40	0.52	EL-Kameesy et al. [21]
India (Karnataka State)	33.23	4.07	0.19	0.29	Chandrashekara et al. [22]
Malaysia	141.62	0.169	0.859	—	Present study
Worldwide	60	0.070	1	—	UNSCEAR [2]

**Table 7:** Comparison of natural radioactivity levels measured in soil in the present study with the values reported in other countries worldwide and established by UNSCEAR [2].

Region/country	Concentration in soil (Bq kg^−1^)						Absorbed dose rates in air (nGy h^−1^)	
	^226^Ra		^232^Th		^40^K			
	Mean	Range	Mean	Range	Mean	Range	Mean	Range
Malaysia	66	49–86	82	63–110	310	170–430		
United States	40	8–160	35	4–130	370	100–700	47	14–118
Japan	33	6–98	28	2–88	310	15–990	53	35–70
China	32	2–440	41	1–360	440	9–1800	62	2–340
India	29	7–81	64	14–160	400	38–760	56	20–1100
Egypt	17	5–64	18	2–96	320	29–650	32	20–133
Iran	28	8–55	22	5–42	640	250–980	71	36–130
Denmark	17	9–29	19	8–30	460	240–610	52	35–70
Spain	32	6–250	33	2–210	470	25–1650	76	40–120
Poland	26	5–120	21	4–77	410	110–970	45	18–97
Switzerland	40	10–900	25	4–70	370	40–1000	45	15–120
Portugal	44	8–65	51	22–100	470	25–1650	76	40–120
Bulgaria	45	12–210	30	7–160	400	40–800	70	48–96
Romania	32	8–60	38	11–75	490	250–1100	59	21–122
Portugal	44	8–65	51	22–100	470	25–1650	76	40–120
Present study	65.24	45.11–111.4	83.39	51.83–127.35	136.98	99.2–172.85	141.62	91.34–207.34
UNSCEAR, 2000	35		30		400		60	53–98
